# The negative effect of joint line elevation after total knee arthroplasty on outcome

**DOI:** 10.1007/s00167-018-5099-8

**Published:** 2018-08-14

**Authors:** Willem A. M. van Lieshout, Kars P. Valkering, Koen L. M. Koenraadt, Faridi S. van Etten-Jamaludin, Gino M. M. J. Kerkhoffs, Rutger C. I. van Geenen

**Affiliations:** 1 Department of Orthopaedic Surgery, Amphia Hospital, Molengracht 21, 4148 CK Breda, The Netherlands; 2 Department of Orthopaedic Surgery, Orthopedium, Delft, The Netherlands; 3 Foundation for Orthopaedic Research, Care and Education, Amphia Hospital, Breda, The Netherlands; 4 Medical Library, Amsterdam UMC, Amsterdam, The Netherlands; 5 Department of Orthopaedic Surgery, Amsterdam UMC, Amsterdam, The Netherlands

**Keywords:** Systematic review, Correlation analysis, Total knee arthroplasty, Revision total knee arthroplasty, Joint line, Outcome

## Abstract

**Purpose:**

Total knee arthroplasty (TKA) is widely used as a treatment for knee osteoarthritis. However, still up to 20% of the patients are dissatisfied. Joint line elevation after TKA might be a contributing factor as it alters knee kinematics. The aim of this study was to investigate the effect of joint line elevation on outcome.

**Methods:**

A systematic review of the literature was performed to select studies that reported on joint line alterations after primary or revision TKA and outcome. Studies with comparable outcome parameters were included in a correlation analysis.

**Results:**

In total, 396 studies were identified, of which 27 met the inclusion criteria. 8 studies could be included in the correlation analysis. Mean joint line elevation after primary TKA was 3.0 mm and after revision TKA this was 3.6 mm. A statistically significant negative correlation was found between joint line elevation and the postoperative Knee Society Score (KSS) function score (*ρ* = − 0.496, *p* < 0.001). In a pooled analysis, the maintained joint line revision TKA group had statistically significant better postoperative KSS total scores compared to an elevated joint line group (*p* < 0.001).

**Conclusion:**

In this systematic review, a negative correlation between joint line elevation and outcome was found. Furthermore, revision TKAs with a maintained joint line have statistically significant better postoperative KSS scores compared to an elevated joint line group. To achieve optimal outcome after TKA, restoration of the joint line is one of the parameters that should be pursued and introduced elevation should not exceed 4 mm.

**Level of evidence:**

IV.

## Introduction

Satisfaction after total knee arthroplasty ranges between 75 and 90% [8, 9, 13, 29]. Different contributing factors for dissatisfaction after TKA have been identified like socioeconomic status, mental well-being, fulfilment of expectation, and the postoperative general physical health of the patient [12]. From a surgical point of view, joint line elevation, among other factors, is considered to have a negative effect on postoperative outcome, since it alters the biomechanics of the knee. By changing the centre of rotation of the knee, the isometry of the medial collateral ligament (MCL) is changed, with mid-flexion instability as a result [15, 34]. In addition, as a consequence of the elevated joint line, the posterior condylar offset (PCO) is likely to be reduced, which negatively influences flexion angle and the extensor mechanism strength, and resolves in mid-flexion instability [6, 36, 37].

Mean joint line elevation after primary TKA varies between 1.1 up to 5.6 mm [44, 46]. For revision TKA, this number is even higher, up to a mean of 8 mm [40]. Some studies report a correlation between a raised joint line and patient-reported outcome measures (PROMs) [10, 17, 40], while others do not find this correlation [49]. Therefore, there is still debate if minor joint line elevation affects PROMs after TKA [44], and if so, what amount of joint line elevation is acceptable after TKA.

Therefore, the aim of the present study was (1) to examine the effect of joint line alteration on outcome measures after primary and revision TKA and (2) to investigate whether practical recommendations can be made concerning acceptable joint line alterations after (revision) TKA.

## Materials and methods

### Identification of studies

A systematic literature search was performed with the help of a clinical librarian. Search terms were: knee arthroplasty, knee replacement, knee surgery, TKA, joint line, outcome, joint instability, knee kinematics, and range of motion. The following databases were searched: PubMed/Medline, the Cochrane Clinical Trial Register, and Embase. The search was limited to English, German, Dutch, and Spanish languages without limitation of publication date. The search was performed August 2017. The reference lists of the included studies were searched for any studies that might potentially meet the inclusion criteria.

### Inclusion and exclusion

PRISMA methodology was used for the analysis and reporting of the systematic review [38]. Titles and abstracts from potentially relevant studies were reviewed using a set of predefined inclusion and exclusion criteria. Studies were included when they reported both joint line alterations after TKA surgery and an outcome measurement. Both primary as well as revision TKA studies were included in our study. Studies reporting on (revision) uni-compartmental knee arthroplasty and knee arthroplasty after high tibial osteotomy (HTO) were excluded.

Two reviewers (WvL and KV) independently screened the titles and abstracts of the studies that could meet the inclusion criteria. A list of studies to be reviewed in full text was composed from the input of both reviewers. Finally, these selected full-text studies were judged on inclusion and exclusion criteria and a definite selection of studies was made. Disagreement in the selection process was debated on and, if necessary, resolved in a group discussion with the third author (KK).

### Data extraction

The following data were extracted from the included studies: number of TKAs, patient demographics, primary or revision TKA, type of prosthesis [cruciate retaining (CR) and posterior stabilized (PS)], method of joint line assessment, mean joint line alteration, outcome scores, and follow-up time. The methodological quality of the included studies was assessed by assigning levels of evidence as defined by the Oxford Centre for Evidence-Based Medicine [22]. Levels of evidence were assigned by two reviewers (WvL and KV). Disagreement was resolved by consensus. A grade of recommendation was added to the present findings based on the methodological quality of the included studies in the systematic review [20].

### Statistical analysis

Statistical analysis was done using IBM SPSS version 24.0. In the analysis, the joint line alteration and TKA outcome scores were weighted by the number of patients. Normal distribution was evaluated by frequency histograms. Degree of correlation [with 95% confidence intervals (CIs)] between mean postoperative joint line alteration and outcome scores was calculated. The preferred test for correlation analysis depends on the distribution of the data; for normally distributed data, Pearson’s correlation is used, for not normally distributed data, Spearman’s rho is used. Significance was set at the 1% (0.01). A small effect size is defined as *ρ* between 0.10 and 0.30, a medium effect size as *ρ* between 0.30 and 0.50, and a large effect size is operationally defined as one that yields *ρ* ≥ 0.50 [14]. An independent-sample t test (confidence interval 95%) was used to compare the outcome of revision TKA groups with the joint line maintained versus elevated.

### Registration

The systematic review was registered in the Prospero Database CRD42017057320.

## Results

**Fig. 1:**
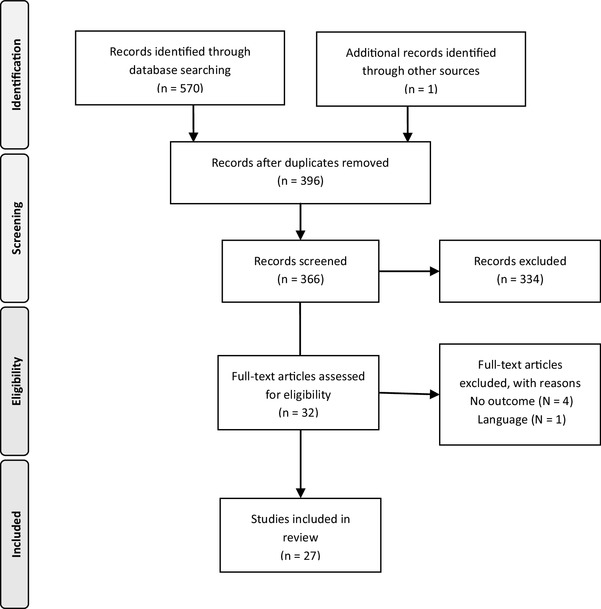
Flowchart systematic review

After performing the search, 396 studies were identified. A total of 27 studies met the inclusion criteria and were included (Fig. 1) [1–4, 7, 10, 11, 17, 19, 21, 23, 25–28, 30, 32, 35, 39–42, 44–47, 49]. These studies were published between 1986 and 2017. A total of 18 studies reported on primary TKA and 9 on revision TKA. The level of evidence for the included studies was level II for 4 studies, level III for 11 studies, and the remaining 12 studies were classified as level IV.

A great diversity was found across papers in the way joint line deviation was measured (Table 1). Revision TKA joint line alteration is defined as the joint line position postoperative compared to either the preoperative or the healthy contralateral knee joint line position. Studies that reported absolute joint line changes were combined to establish a mean joint line change after (r)TKA (see Table 1 for eligible studies). For 1225 primary TKAs, the mean joint line was raised with 3.0 mm. For 355 revision TKAs, the mean joint line elevation was 3.6 mm.

**Table 1:** Study inclusion systematic review: study characteristic table

References	Level of evidence	Study population	Primary/revision	Joint line assessment method	Joint line presentation	Outcome measurement	Follow-up time
Bae et al. [4]	II	32 TKA after HTO 32 TKA	Primary TKA	Fibular head	Absolute values	KSS, WOMAC	6 years
Babazadeh et al. [3]	II	45 MR TKA 49 CAS TKA	Primary TKA	Fibular head Adductor tubercle	Absolute values and subgroups	KSS; SF12; ROM	2 years
Babazadeh et al. [2]	II	52 MR TKA 51 GB TKA	Primary TKA	Resected bone versus prosthesis thickness	Absolute values	KSS; SF12	2 years
Bieger et al. [7]	IV	69 TKA	Revision TKA	Tibial tubercle; epicondyle ratio	Subgroups	KSS	2 years
Bin Abd Razak et al. [1]	III	195 CAS TKA	Primary TKA	Resected bone versus prosthesis thickness	Absolute values	KSS; SF36; OKS; ROM	2 years
Clavé et al. [10]	III	85 TKA	Revision TKA	Fibular head; contralateral healthy side	Absolute values and subgroups	KSS	NA
Clement and MacDonald [11]	III	107 TKA	Revision TKA	Tibial tubercle	No absolute values	OKS, SF12	1 year
Figgie et al. [17]	IV	116 MR TKA	Primary TKA	Tibial tubercle	Absolute values and subgroups	Mayo Clinical Knee Score; ROM	30–60 months
Goh et al. [19]	III	38 CAS TKA 38 iAssist TKA	Primary TKA	Fibular head	Absolute values	KSS; OKS; ROM	6 months
Huang et al. [23]	III	36 MR TKA 34 CAS TKA	Primary TKA	Tibial tubercle	Absolute values	KSS; patella score; ROM	5–10 years
Hofmann et al. [21]	IV	89 TKA	Revision TKA	Adductor tubercle; contralateral side	Absolute values and subgroups	KSS	> 2 years
Ji et al. [25]	III	55 MR TKA	Primary TKA	Adductor tubercle	Subgroups	KSS; patella score; ROM	1 year
Kannan et al. [26]	IV	37 TKA	Revision TKA	Adductor ratio	Absolute values	Modified KSS	NA
Kawamura and Bourne [27]	III	73 TKA	Primary TKA	Fibular head	Absolute values	ROM	2 years
Kazemi et al. [28]	IV	60 TKA	Primary TKA	Blackburn–Peel index	Subgroups	KSS; ROM	2 years
Lee et al. [30]	III	15 NA-MR TKA 15 NA-GB TKA	Primary TKA	Fibular head; tibial tubercle	Absolute values	KSS; ROM	2 years
Liow et al. [32]	II	31 CAS TKA 29 MR TKA	Primary TKA	Fibular head	Absolute values and subgroups	KSS; OKS; SF36; ROM	6 months
Mahoney and Kinsey [35]	IV	22 TKA	Revision TKA	Tibial Tubercle	Absolute values and subgroups	KSS	2 years
Pang et al. [39]	III	100 MR TKA	Primary TKA	Fibular head; tibial tubercle	Absolute values	KSS; OKS; SF36	2 years
Partington et al. [40]	IV	107 TKA	Revision TKA	Tibial tubercle	Absolute values and subgroups	KSS	2 years
Porteous et al. [41]	III	114 TKA	Revision TKA	Tibial tubercle	Subgroups	Bristol Knee Score	1 year
Ryu et al. [42]	IV	90 MR TKA	Primary TKA	Tibial tubercle	Absolute values	ROM	25–90 months
Selvarajah and Hooper [44]	IV	76 TKA	Primary TKA	Tibial tubercle	Absolute values	Modified KSS; ROM	> 30 months
Seon and Song [45]	IV	74 TKA	Revision TKA	Fibular head	Absolute values	KSS; WOMAC	NA
Snider and Macdonald [46]	IV	200 TKA	Primary TKA	Fibular head; tibial tubercle	Absolute values and subgroups	KSS	2 years
Yang et al. [49]	IV	50 CAS TKA	Primary TKA	Resected bone versus prosthesis thickness	Subgroups	KSS; ROM	39–55 months
Vera et al. [47]	III	32 TKA	Primary TKA	Adductor tubercle	Subgroups	KSS; ROM	NA

*MR* measured resection, *TKA* total knee arthroplasty, *HTO* High tibial osteotomy, *CAS* computer-assisted surgery, *GB* gab balancing, *NA* navigation assisted, *KSS* Knee Society Score, *OKS* Oxford Knee Score, *ROM* range of motion, *WOMAC* Western Ontario and McMaster Universities Osteoarthritis Index

Functional assessments were done with the Oxford Knee Score, Short Form 12 (SF-12), Short Form 36 (SF-36), and Knee Society Score (KSS). The KSS is divided in two separate components (knee and function), and some studies reported these two components separately and some presented the score in total. One study used the Mayo Clinical Score, one the Bristol Knee score, and one used the patellar-score [16] to present their results. All studies reported an improvement in clinical scores after TKA. In most studies, the patients were assigned to subgroups with related cut-off values for joint line alterations (i.e., joint line alterations < 4 or > 4 mm). In the group of studies on primary TKA, in 6 out of 18 studies, statistically significant lower outcome scores were found when the joint line was elevated. In the group of studies on revision TKA, five out of nine studies reported statistically significant better outcome scores for patients with an adequately re-created joint line compared to those with an elevated joint line (i.e., joint line reconstruction within 4 mm of the preoperative or healthy contralateral knee joint line height).

**Fig. 2:**
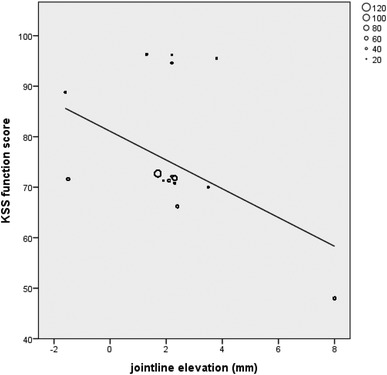
Correlation analysis of KSS function versus joint line alteration in primary TKA

**Fig. 3:**
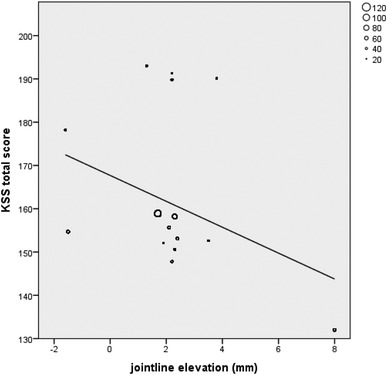
Correlation analysis of KSS total versus joint line alteration in primary TKA

**Fig. 4:**
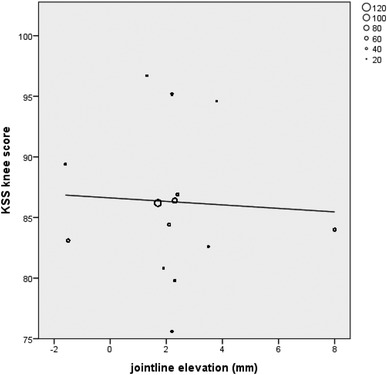
Correlation analysis of KSS knee versus joint line alteration in primary TKA

Eight out of eighteen studies on primary TKA could be included in a correlation analysis of the postoperative KSS knee and function scores and postoperative joint line alteration (mm). The remaining ten studies did not report absolute joint line alteration values or used a different outcome measurement. Seven studies (664 patients) compared two surgical techniques, and in one study (32 patients), one treatment arm could be included in the correlation analysis (Table 2). The other arm contained patients with TKA after HTO which was an exclusion criterion. A statistically significant negative correlation was found between joint line elevation and postoperative KSS functional score (*ρ* = − 0.496, *p* < 0.001, Fig. 2) and KSS total score (*ρ* = − 0.425, *p* < 0.001, Fig. 3). No significant correlation was found between joint line and KSS Knee score (*ρ* = − 0.052, p = 0.17, Fig. 4). Other PROMs presented in the included studies were SF-36 and OKS. Only the study by Liow et al. reported a statistically significant result. In their subgroup analysis (< 5 or > 5 mm joint line shift), the elevated joint line group had a statistically significant lower physical function in the SF-36 questionnaire (45 versus 67) [32]. No significant differences between joint line position subgroups and OKS outcome were reported. The results of the remaining studies that could not be included in the correlation analysis are presented in Table 3. Regarding range of motion (ROM), a wide variety with regards to the notification was observed (i.e., absolute ROM, delta ROM, and only maximum flexion) which limited further analysis. Two studies reported ROM as their sole outcome measurement and, therefore, are not presented in Table 3 [27, 42].

**Table 2:** Studies included for data correlation analysis for primary TKA

References	Number of TKA	Mean JL alteration (mm)^a^	JL assessment method	KSS knee score postop	KSS function score postop
Bae et al. [4]	32	− 1.6	Fibular head	89.4	88.8
Babazadeh et al. [2]	52	− 1.5	Resected bone	83.1	71.6
	51	2.1	Resected bone	84.4	71.3
Bin Abd Razak et al. [1]	112	1.7	Resected bone	86.2	72.7
	83	2.3	Resected bone	86.4	71.8
Goh et al. [19]	38	2.2	Fibular head	75.6	72.2
	38	2.3	Fibular head	79.8	70.8
Huang et al. [23]	34	1.3	Tibial tubercle	96.7	96.3
	36	2.2	Tibial tubercle	95.2	94.6
Lee et al. [30]	30	2.2	Tibial tubercle	95.1	96.2
	30	3.8	Tibial tubercle	94.6	95.5
Liow et al. [32]	31	1.9	Fibular head	80.8	71.3
	29	3.5	Fibular head	82.6	70
Pang et al. [39]	50	2.4	Tibial tubercle	86.9	66.2
	50	8	Tibial tubercle	84	48

*TKA* total knee arthroplasty, *JL* joint line, *KSS* Knee Society Score

^a^Negative values represent joint line decrease; positive values represent joint line elevation

**Table 3:** Studies not included for data correlation analysis for primary TKA

References	Outcome measurement	Joint line method/cut-off value	Findings regarding joint line elevation and outcome
Babazadeh et al. [3]	KSS, SF12	Maintained within 2 mm, depressed of elevated	KSS: Significantly higher changes in KSS total and KSS subscores in maintained joint line group
			SF12: no differences between subgroups
Figgie et al. [17]	Mayo Clinic Knee score	Absolute measurements and < 8 mm or > 8 mm	Negative correlation between joint line elevation and outcome *ρ* − 0.42 (*p* = 0.0001)
Ji et al. [25]	KKS	> 4 mm	Lower scores for > 4 mm joint line elevation compared to the literature
Kazemi et al. [28]	KSS	Pseudo-patella Baja and/or patella baja versus no patella baja	No differences between the subgroups for KSS Total score. Significant lower KSS pain scores for no patella baja group
Selvarajah and Hooper [44]	Modified KSS	Absolute measurement	No statistical correlation between joint line elevation and outcome
Snider and Macdonald [46]	KSS	< 8 mm or > 8 mm	Non-significant better total KSS scores for maintained joint line group (*p* = 0.17)
Yang et al. [49]	KSS	< 3 mm or > 3 mm	No differences between subgroups
Vera et al. [47]	KSS subscales (poor, regular, good, and excellent)	< 4 mm or > 4 mm	Statistically significant better postoperative score for KSS for maintained joint line group

*TKA* total knee arthroplasty, *KSS* Knee Society Score, *SF 12* Short Form 12

Nine studies reported TKA revision outcome data with joint line measurement (Table 4). Indications for revision were infection, aseptic loosening, instability, polyethylene wear, pain, and/or stiffness or peri-prosthetic fracture. No studies reported TKA revision for joint line elevation as its sole reason. Due to heterogeneity of joint line measurements and outcome data, no correlation analysis could be performed. In four studies, patients with a maintained joint line (i.e., < 4, < 5, or < 8 mm joint line elevation) were compared to patients with an elevated joint line after revision TKA. In this analysis, 187 pooled patients in the maintained joint line group were compared with 96 patients in the elevated joint line group [7, 10, 35, 40]. A statistically significant (*p* < 0.001) higher postoperative total KSS score was found for the maintained joint line group (149; SD 10) compared to the elevated joint line group (129; SD 5). Of the five studies that could not be included in the pooled analysis, two studies found better postoperative outcome scores for their maintained joint line group [21, 41] and three studies did not find a significant correlation [11, 26, 45].

**Table 4:** Revision TKA studies

References	JL assessment method	Outcome measurement	Subgroup	Number of rTKA	Outcome KSS total	Knee score postop	Function score Postop	*p* value
Bieger et al. [7]	Medial Epicondyle ratio	KSS	< 5 mm	46	164			< 0.0001
			> 5 mm	23	138			
	Tibial tubercle	KSS	< 5 mm	24	157			n.s.
			> 5 mm	45	155			
Clavé et al. [10]	Fibular head	KSS	< 4 mm	56	142	74	68	KSS Knee n.s. KSS Function 0.014
			> 4 mm	25	130	71	59	
Clement and MacDonald [11]	Tibial tubercle	OKS, SF12	None	107				–
Hofmann et al. [21]	Adductor tubercle	KSS	− 4 to 4 mm	59	–			0.04 between subgroups in favor of restored group
			< − 4 mm or > 4 mm	17	–			
Kannan et al. [26]	Adductor ratio	Modified KSS	–	37				n.s.
Mahoney and Kinsey [35]	Tibial tubercle	KSS	–	22	158	90.7	66.8	n.s.
Partington et al. [40]	Tibial tubercle	KSS	< 8 mm	107 knees in total	141			0.05
			> 8 mm		125			
Porteous et al. [41]	Tibial tubercle	Bristol Knee Score	< 5 mm	73		83.7	20.3	Knee < 0.01
			> 5 mm	41		76.1	17.6	Function < 0.005
Seon and Song [45]	Fibular head	KSS; WOMAC	NR	74				No correlation JL and outcome

*JL* joint line, *rTKA* revision total knee arthroplasty, *NR* not reported, *KSS* Knee Society Score, *WOMAC* Western Ontario and McMaster Universities Osteoarthritis Index

## Discussion

The most important finding of the present study was that joint line elevation has a negative effect on outcome after TKA. A statistically significant negative correlation between joint line elevation and the KSS functional and total score after primary TKA was established. For revision TKA, it was found that a correctly re-created joint line results in statistically significant better postoperative KSS total scores, compared to knees in which the joint line was not adequately re-created.

The statistically significant negative correlation between postoperative joint line elevation and KSS functional scores for primary TKAs is in accordance with several studies that could not be included in the correlation analysis [3, 17, 25, 46, 47]. Four studies did not find a correlation between joint line elevation and outcome scores [1, 2, 19, 49]. These four studies reported only minor mean joint line elevation postoperative (0–2.3 mm). Studies with a larger range in joint line alteration and those who chose a higher cut-off value for joint line analysis did find differences in outcome [17, 32, 46, 47]. In contrast, those with lower cut-off values (e.g., 3 mm) did not find any difference in outcome [3, 49].

Five out of nine studies reporting on revision TKAs found statistically significant better outcome scores for maintained compared to elevated joint line groups [7, 10, 21, 40, 41]. In the pooled analysis of four studies, a statistically significant higher postoperative total KSS score was found for the maintained joint line group compared to the elevated group. This finding was in accordance with two studies that could not be included in this pooled analysis [21, 41]. The studies of Kannan et al. and Clement et al. reported no effect of joint line position on outcome; however, the joint line alteration was not specified [11, 26]. Seon et al. compared two-stage revision TKAs for prosthetic joint infection versus one-stage revision for other reasons. Their subgroup analysis for elevated joint line (> 5 mm) versus the maintained joint line group did not show any differences in outcome scores. However, this result is most likely influenced by the distribution of septic patients amongst their groups since the septic group reported statistically significantly lower outcomes [45].

It is questionable how much joint line elevation can be accepted. Several studies in primary as well as revision TKA have shown statistically significantly lower outcome scores when a cut-off value of 4 mm joint line elevation is exceeded [10, 21, 47]. Babazadeh et al. and Yang et al. used respectively 2 and 3 mm as cut-off point, and they did not find any difference between their groups [3, 49]. Therefore, the aim should be to restore the joint line to its native position and not to accept more than 4 mm of joint line elevation.

Some surgical aspects should be taken into consideration with regards to joint line preservation. First, in case of distal femoral bone loss, the distal femoral bone cut should be reduced. Second, in case of a tight extension gap, it is advised to remove all posterior osteophytes before recutting the distal femur since posterior osteophytes tend to tighter up the posterior capsule and, consequently, reduce the extension gap. Furthermore, computer-assisted surgery and patient-specific instruments can be supportive and promising results with regards to joint line reconstruction are being published [1, 19, 31, 32]. In revision TKA, joint line restoration can be more challenging due to bone loss and the absence of landmarks to determine the original joint line height. Undersizing the femoral component should be avoided and distal and posterior bone loss should be accounted for with augments, thereby restoring the joint line position and the PCO [5]. The three-step technique described by Vince et al. can be a valuable tool to achieve these goals [48]. The medial adductor tubercle is identifiable in most cases, and can be used for preoperative planning and perioperative reference [43]. Preoperative planning, e.g., radiographs of the contralateral knee, can help the surgeon to determine the exact native joint line distance to this landmark before surgery [5].

A major limitation of the current study was the heterogeneity of the data. For both joint line assessment as well as outcome measures, a variety of measurements was reported. This impeded a correlation analysis of the TKA revision studies and of the primary TKA studies only 8 out of 18 primary TKA studies could be included. Furthermore, joint line assessment was performed with a variety of methods which could have influenced the results. However, all reported methods have acceptable inter and intra observer reliability [4, 24, 41], and only absolute changes were used in the analysis, which, in theory, should be interchangeable between the methods. Most of the included studies used the KSS to evaluated patient outcome. It is questionable, however, if this questionnaire is a valid tool to identify symptoms related to joint line alterations (i.e., mid-flexion instability; loss of flexion due to loss of PCO). Functional tests like a 6 min walk test and the stair climbing test have shown to correlate well with mid-flexion instability and might give better insight in functional outcome [18].

Although it was shown by Luyckx et al. that the use of ratio measurements (i.e., medial epicondyle ratio and adductor ratio) is more reliable and reproducible for joint line assessment and reconstruction in revision TKA [33], most commonly the tibial tubercle or the fibular head landmarks are used for evaluating joint line position. These measurements, however, are affected by tibial slope change, and if the joint line is only assessed from the tibial side, femoral sided joint line alterations are unrecognized. Therefore, the ideal joint line assessment would include a separate tibial and femoral referenced measurement. Since only one primary TKA [3] and one revision TKA study [7] reported separate data for the femoral and tibial sided joint line alterations, a separate tibial and femoral analysis could not be performed. Interestingly, both studies illustrated discrepancies between tibial and femoral based joint line measurements. However, correlations of these measurements with outcome scores showed conflicting results. Due to the relatively low level of evidence of the included studies, a weak recommendation can be assigned to the suggested cut-off value of 4 mm maximal acceptable joint line elevation.

## Conclusion

In this systematic review, a negative correlation between joint line elevation and outcome was found, with an elevation of more than 4 mm resulting in statistically significant lower outcome scores. Hence, it is advised not to exceed 4 mm of joint line elevation in primary TKA. For revision TKAs, the aim should be to restore the joint line to its native height.

## Abbreviations

CAS: Computer-assisted surgery
CR: Cruciated retaining
GB: Gap balancing
HTO: High tibial osteotomy
JL: Joint line
KSS: Knee society score
MR: Measured resection
NA: Navigation assisted
OKS: Oxford knee score
PCO: Posterior condylar offset
PROMs: Patient-reported outcome measurements
PS: Posterior stabilized
ROM: Range of motion
SF 12/36: Short Form
rTKA: Revision total knee arthroplasty
TKA: Total knee arthroplasty
WOMAC: Western Ontario and McMaster Universities Osteoarthritis Index

## References

[1] Bin Abd Razak HR, Pang HN, Yeo SJ, Tan MH, Lo NN, Chong HC (2013). Joint line changes in cruciate-retaining versus posterior-stabilized computer-navigated total knee arthroplasty. Arch Orthop Trauma Surg.

[2] Babazadeh S, Dowsey MM, Stoney JD, Choong PFM (2014). Gap balancing sacrifices joint-line maintenance to improve gap symmetry: a randomized controlled trial comparing gap balancing and measured resection. J Arthroplast.

[3] Babazadeh S, Dowsey MM, Swan JD, Stoney JD, Choong PFM (2011). Joint line position correlates with function after primary total knee replacement: a randomised controlled trial comparing conventional and computer-assisted surgery. J Bone Jt Surg Br.

[4] Bae DK, Song SJ, Park CH, Liang H, Bae JK (2017). Comparison of mid-term results between conversion total knee arthroplasties following closed wedge high tibial osteotomy and primary total knee arthroplasties: a matched pair study including patellar symptom and position. J Orthop Sci.

[5] Bellemans J (2004). Restoring the joint line in revision TKA: does it matter?. Knee.

[6] Bellemans J, Banks S, Victor J, Vandenneucker H, Moemans a (2002). Fluoroscopic analysis of the kinematics of deep flexion in total knee arthroplasty. Influence of posterior condylar offset. J Bone Jt Surg Br.

[7] Bieger R, Huch K, Kocak S, Jung S, Reichel H, Kappe T (2014). The influence of joint line restoration on the results of revision total knee arthroplasty: comparison between distance and ratio-methods. Arch Orthop Trauma Surg.

[8] Bourne RB, Chesworth BM, Davis AM, Mahomed NN, Charron KDJ (2010). Patient satisfaction after total knee arthroplasty: who is satisfied and who is not?. Clin Orthop Relat Res.

[9] Choi Y-J, Ra HJ (2016). Patient satisfaction after total knee arthroplasty. Knee Surg Relat Res.

[10] Clavé A, Le Henaff G, Roger T, Maisongrosse P, Mabit C, Dubrana F (2016). Joint line level in revision total knee replacement: assessment and functional results with an average of seven years follow-up. Int Orthop.

[11] Clement N, MacDonald D (2017). Posterior condylar offset is an independent predictor of functional outcome after revision total knee arthroplasty. J Bone Jt Surg.

[12] Clement ND (2013). Patient factors that influence the outcome of total knee replacement: a critical review of the literature. Open Access Orthop.

[13] Clement ND, Bardgett M, Weir D, Holland J, Deehan DJ (2018). Increased symptoms of stiffness 1 year after total knee arthroplasty are associated with a worse functional outcome and lower rate of patient satisfaction. Knee Surg Sport Traumatol Arthrosc.

[14] Cohen J (1988). Statistical power analysis for the behavioral sciences.

[15] Cross MB, Nam D, Plaskos C, Sherman SL, Lyman S, Pearle AD, Mayman DJ (2012). Recutting the distal femur to increase maximal knee extension during TKA causes coronal plane laxity in mid-flexion. Knee.

[16] Feller J, Bartlett RJ, Lang DM (1996). Patellar resurfacing versus retention in total knee arthroplasty. J Bone Jt Surg Br.

[17] Figgie HE, Goldberg VM, Heiple KG, Moller HS, Gordon NH (1986). The influence of tibial–patellofemoral location on function of the knee in patients with the posterior stabilized condylar knee prosthesis. J Bone Jt Surg Am.

[18] Freisinger GM, Hutter EE, Lewis J, Granger JF, Glassman AH, Beal MD, Pan X, Schmitt LC, Siston RA, Chaudhari AMW (2017). Relationships between varus–valgus laxity of the severely osteoarthritic knee and gait, instability, clinical performance, and function. J Orthop Res.

[19] Goh GSH, Liow MHL, Lim WSR, Tay DKJ, Yeo SJ, Tan MH (2016). Accelerometer-based navigation is as accurate as optical computer navigation in restoring the joint line and mechanical axis after total knee arthroplasty. A prospective matched study. J Arthroplast.

[20] Guyatt GH, Oxman AD, Kunz R, Falck-Ytter Y, Vist GE, Liberati A, Schunemann HJ (2008). Going from evidence to recommendations. BMJ.

[21] Hofmann AA, Kurtin SM, Lyons S, Tanner AM, Bolognesi MP (2006). Clinical and radiographic analysis of accurate restoration of the joint line in revision total knee arthroplasty. J Arthroplast.

[22] Howick J, Chalmers I, Glasziou P, Greenhalgh T, Heneghan C, Liberati A, Hodgkinson M (2011). The Oxford 2011 levels of evidence.

[23] Huang T-W, Lee C-Y, Lin S-J, Peng K-T, Huang K-C, Lee MS, Hsu RW-W, Shen W-J (2014). Comparison of computer-navigated and conventional total knee arthroplasty in patients with Ranawat type-II valgus deformity: medium-term clinical and radiological results. BMC Musculoskelet Disord.

[24] Iacono F, Lo Presti M, Bruni D, Raspugli GF, Bignozzi S, Sharma B, Marcacci M (2013). The adductor tubercle: a reliable landmark for analysing the level of the femorotibial joint line. Knee Surg Sport Traumatol Arthrosc.

[25] Ji S-J, Zhou Y-X, Jiang X, Cheng Z-Y, Wang G-Z, Ding H, Yang M-L, Zhu Z-L (2015). Effect of joint line elevation after posterior-stabilized and cruciate-retaining total knee arthroplasty on clinical function and kinematics. Chin Med J.

[26] Kannan A, O’Connell RS, Kalore N, Curtin BM, Hull JR, Jiranek WA (2015). Revision TKA for flexion instability improves patient reported outcomes. J Arthroplast.

[27] Kawamura H, Bourne RB (2001). Factors affecting range of flexion after total knee arthroplasty. J Orthop Sci.

[28] Kazemi SM, Daftari Besheli L, Eajazi A, Miniator Sajadi MR, Okhovatpoor MA, Farhang Zanganeh R, Minaei R (2011). Pseudo-patella baja after total knee arthroplasty. Med Sci.

[29] Lange JK, Lee YY, Spiro SK, Haas SB (2018). Satisfaction rates and quality of life changes following total knee arthroplasty in age-differentiated cohorts. J Arthroplast.

[30] Lee HJ, Lee JS, Jung HJ, Song KS, Yang JJ, Park CW (2011). Comparison of joint line position changes after primary bilateral total knee arthroplasty performed using the navigation-assisted measured gap resection or gap balancing techniques. Knee Surg Sports Traumatol Arthrosc.

[31] Lee K-J, Moon J-Y, Song E-K, Lim H-A, Seon J-K (2012). Minimum two-year results of revision total knee arthroplasty following infectious or non-infectious causes. Knee Surg Relat Res.

[32] Liow MHL, Xia Z, Wong MK, Tay KJ, Yeo SJ, Chin PL (2014). Robot-assisted total knee arthroplasty accurately restores the joint line and mechanical axis. A prospective randomised study. J Arthroplast.

[33] Luyckx T, Beckers L, Colyn W, Vandenneucker H, Bellemans J (2014). The adductor ratio: a new tool for joint line reconstruction in revision TKA. Knee Surg Sports Traumatol Arthrosc.

[34] Luyckx T, Vandenneucker H, Ing LS, Vereecke E, Ing AV, Victor J (2018). Raising the joint line in TKA is associated with mid-flexion laxity: a study in cadaver knees. Clin Orthop Relat Res.

[35] Mahoney OM, Kinsey TL (2006). Modular femoral offset stems facilitate joint line restoration in revision knee arthroplasty. Clin Orthop Relat Res U S.

[36] Martin JW, Whiteside LA (1990). The influence of joint line position on knee stability after condylar knee arthroplasty. Clin Orthop Relat Res.

[37] Matziolis G, Brodt S, Windisch C, Roehner E (2017). Changes of posterior condylar offset results in midflexion instability in single-radius total knee arthroplasty. Arch Orthop Trauma Surg.

[38] Moher D, Liberati A, Tetzlaff J, Altman DG (2009). Reprint–preferred reporting items for systematic reviews and meta-analyses: the PRISMA statement. Phys Ther.

[39] Pang H-N, Yeo S-J, Chong H-C, Chin P-L, Chia S-L, Lo N-N (2013). Joint line changes and outcomes in constrained versus unconstrained total knee arthroplasty for the type II valgus knee. Knee Surg Sports Traumatol Arthrosc.

[40] Partington PF, Sawhney J, Rorabeck CH, Barrack RL, Moore J (1999). Joint line restoration after revision total knee arthroplasty. Clin Orthop Relat Res.

[41] Porteous AJ, Hassaballa MA, Newman JH (2008). Does the joint line matter in revision total knee replacement?. J Bone Jt Surg Br.

[42] Ryu J, Saito S, Yamamoto K, Sano S (1993). Factors influencing the postoperative range of motion in total knee arthroplasty. Bull Hosp Jt Dis.

[43] Sadaka C, Kabalan Z, Hoyek F, Abi Fares G, Lahoud JC (2015). Joint line restoration during revision total knee arthroplasty: an accurate and reliable method. Springerplus.

[44] Selvarajah E, Hooper G (2009). Restoration of the joint line in total knee arthroplasty. J Arthroplast.

[45] Seon J-K, Song E-K (2016). Joint line and patellar height restoration after revision total knee arthroplasty. Indian J Orthop.

[46] Snider MG, Macdonald SJ (2009). The influence of the posterior cruciate ligament and component design on joint line position after primary total knee arthroplasty. J Arthroplast.

[47] Vera-Aviles FA, Jimenez-Aquino JM (2012). Total knee arthroplasty. Prognosis after restoring the joint line. Acta Ortop Mex.

[48] Vince KG, Droll K, Chivas D (2008). New concepts in revision total knee arthroplasty. J Surg Orthop Adv.

[49] Yang J-H, Seo J-G, Moon Y-W, Kim M-H (2009). Joint line changes after navigation-assisted mobile-bearing TKA. Orthopedics.

